# Feasibility of function‐guided lung treatment planning with parametric response mapping

**DOI:** 10.1002/acm2.13436

**Published:** 2021-10-26

**Authors:** Charles K. Matrosic, D. Rocky Owen, Daniel Polan, Yilun Sun, Shruti Jolly, Caitlin Schonewolf, Matthew Schipper, Randall K. Ten Haken, Craig J. Galban, Martha Matuszak

**Affiliations:** ^1^ Department of Radiation Oncology University of Michigan Ann Arbor Michigan USA; ^2^ School of Public Health University of Michigan Ann Arbor Michigan USA; ^3^ Department of Radiology University of Michigan Ann Arbor Michigan USA

**Keywords:** functional imaging, lung cancer, treatment planning

## Abstract

**Purpose:**

Recent advancements in functional lung imaging have been developed to improve clinicians’ knowledge of patient pulmonary condition prior to treatment. Ultimately, it may be possible to employ these functional imaging modalities to tailor radiation treatment plans to optimize patient outcome and mitigate pulmonary complications. Parametric response mapping (PRM) is a computed tomography (CT)–based functional lung imaging method that utilizes a voxel‐wise image analysis technique to classify lung abnormality phenotypes, and has previously been shown to be effective at assessing lung complication risk in diagnostic applications. The purpose of this work was to demonstrate the implementation of PRM guidance in radiotherapy treatment planning.

**Methods and materials:**

A retrospective study was performed with 18 lung cancer patients to test the incorporation of PRM into a radiotherapy planning workflow. Paired inspiration/expiration pretreatment CT scans were acquired and PRM analysis was utilized to classify each voxel as normal, parenchymal disease, small airway disease, and emphysema. Density maps were generated for each PRM classification to contour high density regions of pulmonary abnormalities. Conventional volumetric‐modulated arc therapy and PRM‐guided treatment plans were designed for each patient.

**Results:**

PRM guidance was successfully implemented into the treatment planning process. The inclusion of PRM priorities resulted in statistically significant (*p* < 0.05) improvements to the V20Gy within the PRM avoidance contours. On average, reductions of 5.4% in the V20Gy(%) were found. The PRM‐guided treatment plans did not significantly increase the dose to the organs at risk or result in insufficient planning target volume coverage, but did increase plan complexity.

**Conclusions:**

PRM guidance was successfully implemented into a treatment planning workflow and shown to be effective for dose redistribution within the lung. This work has provided a framework for the potential clinical implementation of PRM‐guided treatment planning.

## 1 INTRODUCTION

Lung cancer is one of the most diagnosed subtypes of cancer and is a leading cause of death. In fact, an estimated 228 820 new cases and 135 720 deaths were recorded in the United States in 2020.^1^ Moreover, 60% of these patients will require radiotherapy over the course of their treatment,^2^, ^3^ and lung cancer is often comingled with various forms of pulmonary comorbidities, such as chronic obstructive pulmonary disease (COPD), due to shared risk factors.^4^, ^5^ Despite the common prevalence of these pulmonary comorbidities, which are known to be poor prognostic factors, there is currently no standard technique to identify and quantify pulmonary diseases prior to radiation therapy.

One strategy that has recently been investigated to mitigate pulmonary complications is to personalize a patient's treatment plan based on underlying lung function. Various forms of functional lung imaging, such as single photon‐emission computed tomography (SPECT), four‐dimensional computed tomography (4DCT), and hyperpolarized Xe magnetic resonance imaging, have been employed to quantify the ability of local lung parenchyma to successfully perform gas transfer, which is the ultimate determinant of a functional lung.^6^ By acquiring this information, these imaging modalities allow for the potential to personalize a patient's treatment plan such that the functional damage is minimized, and thus, it may be possible to reduce radiation‐induced lung toxicity (RILT) rates.^6^ As such, “function‐guided treatment planning” allows for treatment plans to selectively avoid radiosensitive regions of the lung.^7^ Traditionally, functional regions of the lung have been avoided to spare the relatively healthy areas, which generally results in the dose being preferentially funneled through defected regions due to their inability to reperfuse after radiotherapy.^8^, ^9^, ^10^ Based on this theory, functional‐avoidance approaches have been investigated in retrospective studies and are also currently being implemented into prospective clinical trials.^11^, ^12^, ^13^, ^14^ While this technique may be beneficial in some cases, a strong correlation between pulmonary comorbidities and RILT suggests that patients with underlying pulmonary diseases may be at a higher risk for toxicity even when treated with lower radiation doses.^15^, ^16^, ^17^ Furthermore, some recent studies have found that a lower functioning lung appears inherently more susceptible to radiation.^15^, ^18^ The works of both Otsuka et al. and Owen et al. found that the volume receiving greater than or equal to 20 Gy (V20Gy) in regions of poor lung function showed high predictive power of a patient's RILT risk, suggesting that the dose to low function regions should be mitigated.

Over the past decade, computed tomography (CT) has gained high interest for imaging functional lungs. CT‐based functional lung imaging typically utilizes either a 4DCT or paired inhale/exhale scans for use in mapping biomechanical ventilation. By spatially aligning 4DCT phases, or paired CTs, to a single geometric frame by deformable registration, the change in Hounsfield Units (HU) of voxels can be directly compared between phases. This allows one to spatially resolve the propensity for local ventilation. Since each patient is already required to have a treatment simulation CT scan, these CT‐based methods have proven desirable for radiotherapy applications. In one of the most advanced clinical trials, CT‐based function‐guided treatment planning was successfully implemented as part of a phase 2 trial and was shown to improve the V20Gy(%) to a functional lung by 3.2% compared to standard retreatments, which resulted in an estimated reduction in grade ≥2 toxicity from 25% to 17.6%.^11^

A novel method of CT‐based functional lung imaging is parametric response mapping (PRM), which was introduced by Galban et al. in 2012. PRM is unique in that it is a CT imaging voxel‐wise analysis technique for simultaneously assessing multiple pulmonary abnormality phenotypes, such as the small airway disease (SAD) and emphysema, which are key components of COPD.^19^ This method utilizes paired inspiration and expiration CT scans to assign classifications to each lung voxel that represent four subsets of lung parenchyma: normal, emphysema, SAD, and parenchymal disease (PD). Along with being identified as a useful biomarker for COPD, PRM has been shown to be effective at identifying bronchitis obliterans syndrome risk after hematopoietic stem cell transplantation, emphysema development risk in smokers, and fibrosis in lung transplant patients.^20^, ^21^, ^22^ Recent work has extended the use of PRM to investigate its ability as a screening tool in treatment planning and risk assessment for lung cancer patients undergoing radiotherapy.^23^, ^24^

The purpose of this work was to implement PRM guidance into a clinical lung treatment planning workflow. The feasibility of PRM‐guided treatment planning was tested by comparing PRM‐guided treatment plans to standard plans to investigate whether the PRM guidance could effectively reduce the dose to diseased regions without degrading the quality of the treatment plan. To the authors’ knowledge, this is the first study utilizing PRM functional lung imaging to guide radiotherapy treatment planning.

## 2 METHODS AND MATERIALS

A retrospective treatment planning study was designed with the goal of investigating whether PRM guidance could be implemented into a clinical treatment planning workflow, and secondarily, confirming that the inclusion of the PRM data could reduce dose metrics in avoidance contours without violating higher priority treatment planning goals. This study design was accomplished by creating and comparing two treatment plans for a cohort of lung cancer patients: a conventional volumetric‐modulated arc therapy (VMAT) treatment plan and a PRM‐guided VMAT treatment plan.

The patient cohort consisted of 18 lung and mediastinal cancer patients from an institutional clinical trial. The original aim of this clinical trial was to investigate the utility of novel noninvasive imaging biomarkers to identify pathologic changes in pulmonary and cardiac function for thoracic cancer patients. Ideally, these imaging biomarkers could then be used prospectively to assist in treatment plan dose optimization and lead to other therapeutic interventions to reduce cardiopulmonary toxicity. A total of 14 of the patients had 4DCT‐based simulations with their treatment plan created using the untagged 4DCT. In the remaining four cases, the treatment planning CT scan was acquired during deep‐inspiration breath hold (DIBH) assisted by the SDX respiratory gating system (DYN'R, Aix‐en‐Provence, France). Selection of DIBH over 4DCT depended on the patient's ability to perform DIBH and also the potential of dosimetric improvement with the utilization of DIBH. As part of this clinical trial, and in addition to the acquisition of patient treatment planning CTs, paired inspiration/expiration thoracic CT scans were obtained for PRM metric calculation.

**FIGURE 1:**
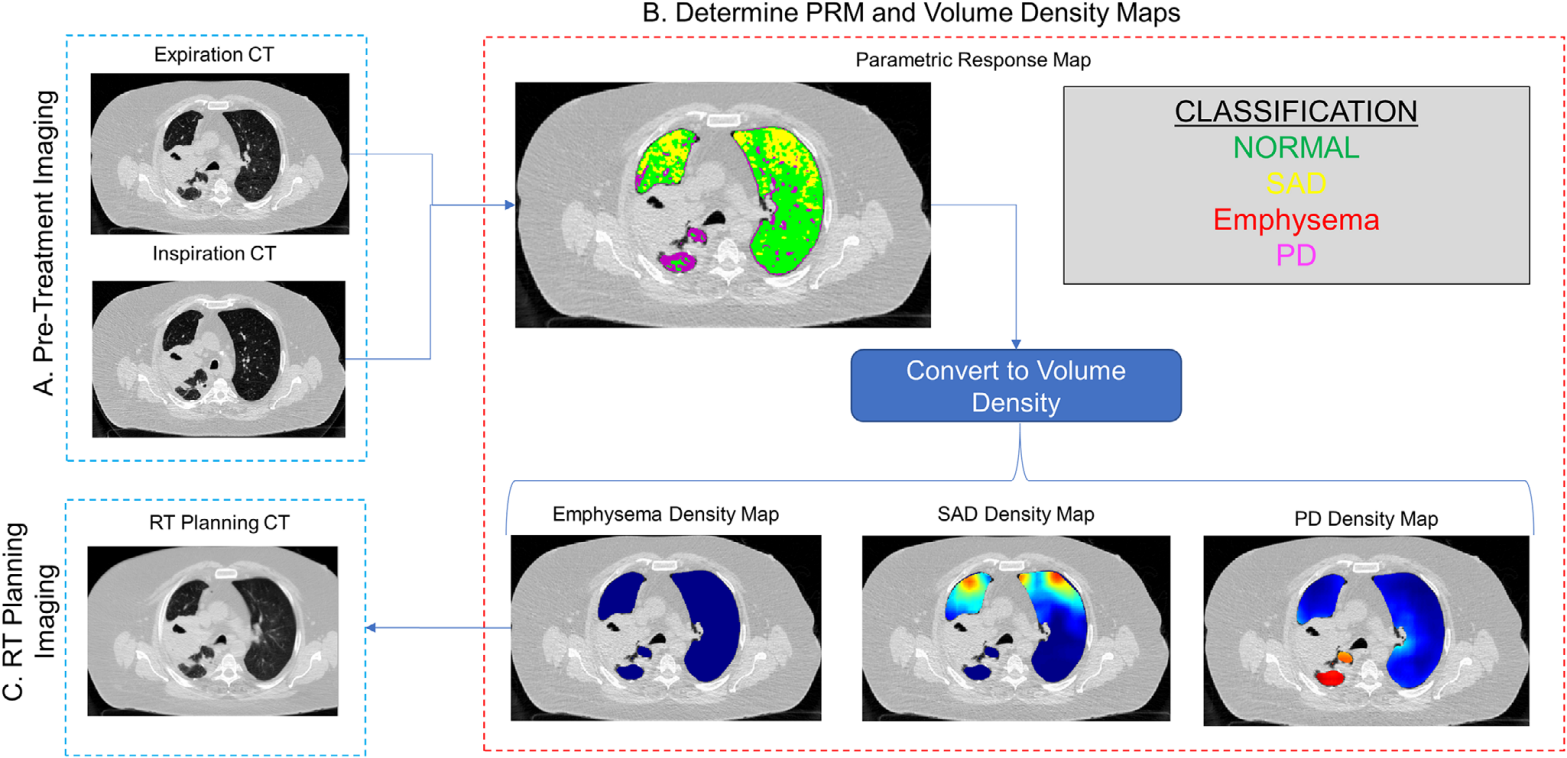
Integration of parametric response mapping into a radiotherapy treatment planning workflow for treating locally advanced lung cancer patients

The inspiration and expiration CT scans were processed using the PRM method previously described by Galban et al.,^21^, ^25^ and is demonstrated in Figure 1. Following segmentation of the lungs from the thoracic cavity, deformable image registration (DIR) was performed using an open‐source software application (Elastix) to align the inspiration CT scan to the expiration CT geometric frame.^26^, ^27^ The result of this process is to associate the voxel densities, measured in HU, to both inflation levels. The inspiration and expiration HU values of each voxel were recorded and categorized based on the PRM classification scheme.^21^ The output of PRM is a 3D map derived in the frame of reference of the expiration CT that classifies individual voxels as normal, SAD, emphysema, and PD. Categorical PRM maps were further processed to generate volume density maps for each PRM classification using the methods previously described by Hoff et al.^25^ In brief, PRM class volume density maps were generated using a moving window of 21 × 21 × 21 voxels approach. The volume density within the window was defined as the number of like PRM class normalized to the sum of all voxels. All PRM processing was performed using in‐house algorithms developed in a technical computing language (MATLAB v. 2019a, The MathWorks Inc., Natick, MA).

Volume density maps of each PRM abnormal function classification were contoured in the Eclipse Treatment Planning System (TPS) (Varian Medical Systems, Palo Alto, CA) using a volume density threshold greater than 0.2 for SAD and PD and greater than 0.1 for emphysema.^19^, ^20^ To utilize the PRM contours in treatment planning, the expiration scan used to calculate the PRM data was registered to the patient's treatment planning simulation 4DCT or DIBH CT. A rigid registration of the exhale scan to either a mid‐breathing cycle phase of the 4DCT or the DIBH CT was first attempted. For free‐breathing cases, a mid‐cycle phase that most closely matched the untagged 4DCT was selected for this registration to reduce the potential effects of motion artifacts on the registration process. In cases where the rigid registration was visually insufficient, B‐spline‐based DIR was performed to improve alignment using Velocity 4.1 (Varian Medical Systems). For these registrations, the extended deformable multipass algorithm was used with the contrast setting focused on lung parenchyma. This DIR method was previously validated following the processes outlined by American Association of Physicists in Medicine (AAPM) Task Group (TG) 132 with the DIR‐Lab thoracic 4DCT dataset and was found to provide accuracy similar to or better than that of other commercially available DIR algorithms with interpatient average three‐dimensional registration error of 2.2 mm (range 1.9–2.6 mm).^28^, ^29^, ^30^, ^31^ In this study, a qualitative evaluation of each DIR was performed based on the visual examination of the anatomical overlap as well as a review of the deformation vector field, DIR animation, and Jacobian map to ensure that the registrations were physiologically plausible. The resulting registration matrix or DIR vector field was used to propagate the PRM contours into alignment with the treatment planning CT.

For the purpose of this retrospective study, all patient treatments were replanned as full‐arc conventional VMAT plans using Eclipse. The treatment plans were created with a prescribed dose of 60 Gy in 30 fractions. The Priority 1 and Priority 2 planning goals were based on current clinical planning goals and are shown in Table 1. Note that the Priority 3 and Priority 4 PRM‐related goals were not included in these conventional plans. Initial optimization cost functions were created using a lung treatment knowledge‐based planning model trained using 43 lung patients from the Michigan Radiation Oncology Quality Consortium (MROQC). Cost functions were modified after the initial optimization to meet planning goals. Dose distributions were calculated using the anisotropic analytical algorithm (AAA) model.^32^

**TABLE 1:** Treatment planning goals during this study

Structure	Priority	Parameter planning goal
Lungs‐GTV or lungs‐IGTV	1	Mean (Gy) ≤ 20 Gy
		V20Gy (%) ≤ 35%
Esophagus	1	D0.03cc (Gy) ≤ 105% Rx dose
		D2.0cc (Gy) ≤ 68 Gy
		Mean (Gy) ≤ 34 Gy
Heart	1	D0.03cc (Gy) ≤ 105% Rx dose
		Mean (Gy) ≤ 20 Gy
		V30Gy (%) ≤ 50%
		V50Gy (%) ≤ 25%
Spinal canal	1	D0.03cc (Gy) ≤ 45 Gy
Spinal canal PRV	1	D0.03cc (Gy) ≤ 50 Gy
Brachial plexus	1	D0.03cc (Gy) ≤ 66.00
PTV	2	D95% (%) ≥ 100% Rx dose
		DC0.1cc (%) ≥ 93% Rx dose
		D0.1cc (%) ≤ 110% Rx dose
PRM SAD*	3	Minimize V20Gy (%)
PRM PD*	3	Minimize V20Gy (%)
PRM emphysema*	4	Minimize V20Gy (%)

* Priority 3 and 4 goals were only utilized in PRM‐guided treatment plans.

Abbreviations: GTV, gross tumor volume; IGTV, internal gross tumor volume; PRV, planning organ at risk volume; PTV, planning target volume; PRM, parametric response mapping, SAD, small airway disease, PD, parenchymal disease.

A second, PRM‐guided, treatment plan was created for each patient using the same initial cost function and beam arrangement as that of the conventional plan. Goals for the PRM density contours of the SAD, PD, and emphysema regions were added to the conventional treatment plan cost function with the aim of reducing the V20Gy of each of the contours without violating any planning goals, as shown in Table 1. Reduction of the SAD and PD V20Gy was prioritized over the emphysema V20Gy because the patients had minimal emphysema volume density. Reduction of V20Gy in the abnormal lung regions was selected as a priority based on the work of Owen et al.^15^ and the theory that pulmonary comorbidities may be exacerbated by a high dose. However, it should be noted that this workflow could also easily be inverted to avoid dose to normal‐functioning lung (i.e., PRM‐defined normal as shown in Figure 1) if desired. Note that in these treatment plans, reduction of the lungs‐gross tumor volume (GTV) doses was a higher priority than PRM avoidance contour priority, therefore dose was not necessarily funneled from the avoidance structures to the healthy lung tissue. If a Priority 2 goal was violated in the conventional plan as a compromise to maintain the Priority 1 goals, the dose volume histogram (DVH) metric of the Priority 2 goal was not allowed to vary by more than 1% from the conventional treatment plan in the PRM‐guided plan. The DVH metrics listed in Table 1 were compared between the PRM‐guided plans and the conventional plans for each patient in the 18‐patient cohort through the calculation of the difference between the values for each plan. The averages, standard deviations (SDs), and confidence intervals of the differences were calculated for each DVH metric. *p*‐values were calculated for the DVH metric differences using a Wilcox signed‐rank test adjusted for multiple testing with the Benjamini–Hochberg procedure.

## 3 RESULTS

Conventional and PRM‐guided treatment plans were successfully created for all 18 patients. DIR was required in 11 cases to provide better alignment between the PRM and planning scans. For the other seven cases, we determined that using DIR was more likely to introduce additional uncertainties rather than resolve minor spatial differences, particularly in the presence of motion artifacts for free‐breathing cases. The relative and absolute V20Gy volumes of the PRM contours for the conventional and PRM‐guided treatment plans were recorded. The difference between the values was calculated for each patient, subtracting the conventional V20Gy from the PRM‐guided V20Gy, and summary statistics of these improvements are shown in Table 2. All treatment plans resulted in either the same or lower V20Gy volumes in the PRM‐guided plans. These improvements were found to be statistically significant (*p* < 0.05). The small absolute volumes of the emphysema contours caused small improvements in the absolute V20Gy(cc) to cause large improvements of the relative V20Gy(%), which is reflected in the large average improvement and SD. The V20Gy volumes of all of the PRM avoidance contours on average were improved by 5.4% and 29.41cc in the PRM‐guided plans compared to the conventional plans.

**TABLE 2:** The average, standard deviation, confidence interval, and adjusted *p*‐values of the parametric response mapping (PRM) contour dose metric changes between the conventional and PRM‐guided treatment plans; note that change in a dose metric was calculated as the conventional plan value subtracted from the PRM‐guided plan value, meaning a negative value signifies a lower value in the PRM‐guided plan

Classification	Metric	Average	Standard deviation	95% CI lower boundary	95% CI upper boundary	*p*‐value
Emphysema	V20Gy (%)	−8.95	17.21	−20.37	2.47	0.014
Emphysema	V20Gy (cc)	−1.17	2.44	−2.79	0.45	0.014
SAD	V20Gy (%)	−4.90	4.43	−7.17	−2.63	<0.001
SAD	V20Gy (cc)	−36.75	46.80	−60.70	−12.80	<0.001
PD	V20Gy (%)	−3.42	3.30	−5.10	−1.73	<0.001
PD	V20Gy (cc)	−37.24	52.37	−64.04	−10.44	<0.001

Abbreviations: SAD, small airway disease; PD, parenchymal disease.

Organ‐at‐risk (OAR) and planning target volume (PTV) dose metrics were recorded and the differences between the conventional and PRM‐guided plans were calculated, with the summary statistics of these changes shown in Table 3. Generally, the plans showed minimal change in the PTV and OAR doses due to the redistribution of the dose away from the regions SAD, PD, and emphysema. These changes were not found to be statistically significant (*p* > 0.05). This often was due to the redistribution of dose to regions of fat and muscle. It was noted that this redistribution did result in a slight decrease in lungs‐GTV V20Gy and mean dose, which were both found to be statistically significant changes (*p* < 0.05). In a few specific cases, a D0.03cc increase in the spinal cord and its planning OAR volume was observed. This occurred when the conventional plan resulted in D0.03cc doses well below the dose limit, and the PRM guidance resulted in dose redistribution to the spinal cord region that increased the D0.03cc by 1–3 Gy while staying below the dose limit.

**TABLE 3:** The average, standard deviation, confidence interval, and adjusted *p*‐values of the organ‐at‐risk and planning target volume (PTV) dose metric changes between the conventional and parametric response mapping (PRM)–guided treatment plans; note that change in a dose metric was calculated as the conventional plan value subtracted from the PRM‐guided plan value, meaning a negative value signifies a lower value in the PRM‐guided plan

ROI	Metric	Average	Standard deviation	95% CI lower boundary	95% CI upper boundary	*p*‐value
L brachial plexus	D0.03cc (Gy)	−0.13	1.23	−1.55	1.29	1
R brachial plexus	D0.03cc (Gy)	−0.06	0.33	−0.52	0.40	1
Esophagus	D0.03cc (%)	0.24	2.80	−1.20	1.67	1
Esophagus	D2cc (Gy)	−0.15	1.52	−0.93	0.62	1
Esophagus	Mean (Gy)	−0.09	0.56	−0.38	0.20	1
Heart	D0.03cc (%)	−0.20	0.50	−0.46	0.05	0.575
Heart	Mean (Gy)	0.00	0.36	−0.19	0.18	1
Heart	V30Gy (%)	0.06	1.03	−0.47	0.58	
Heart	V50Gy (%)	0.06	0.34	−0.11	0.24	1
Heart	V5Gy (%)	−0.79	1.61	−1.62	0.03	0.486
Lungs‐GTV	Mean (Gy)	−0.31	0.37	−0.49	−0.12	0.002
Lungs‐GTV	V20Gy (%)	−1.85	2.13	−2.94	−0.76	0.003
Lungs‐GTV	V5Gy (%)	0.04	0.52	−0.22	0.31	1
PTV	D95% (%)	−0.03	0.21	−0.42	0.11	0.621
PTV	DC0.1cc (%)	−0.22	0.94	−0.14	0.08	1
PTV	D0.1cc (%)	−0.16	0.51	−0.70	0.26	1
SpinalCord	D0.03cc (Gy)	0.60	1.86	−0.35	1.56	0.575
SpinalCord_PRV	D0.03cc[Gy]	0.49	1.34	−0.20	1.17	0.633

Abbreviations: GTV, gross tumor volume; PRV, planning organ at risk volume; PTV, planning target volume.

It was observed that the inclusion of PRM guidance increased the complexity of the plans. On average, the aperture‐based edge metric increased 6.6% (SD: 7.3%), with a maximum increase of 24% and 13 of the 18 cases resulting in increases greater than 2%.^33^ This was also reflected by a 4.6% (SD: 5.3%) increase in planned monitor units (MUs). Despite these increases, the complexities of the plans for all 18 patients were well within deliverable values previously observed clinically.

**FIGURE 2:**
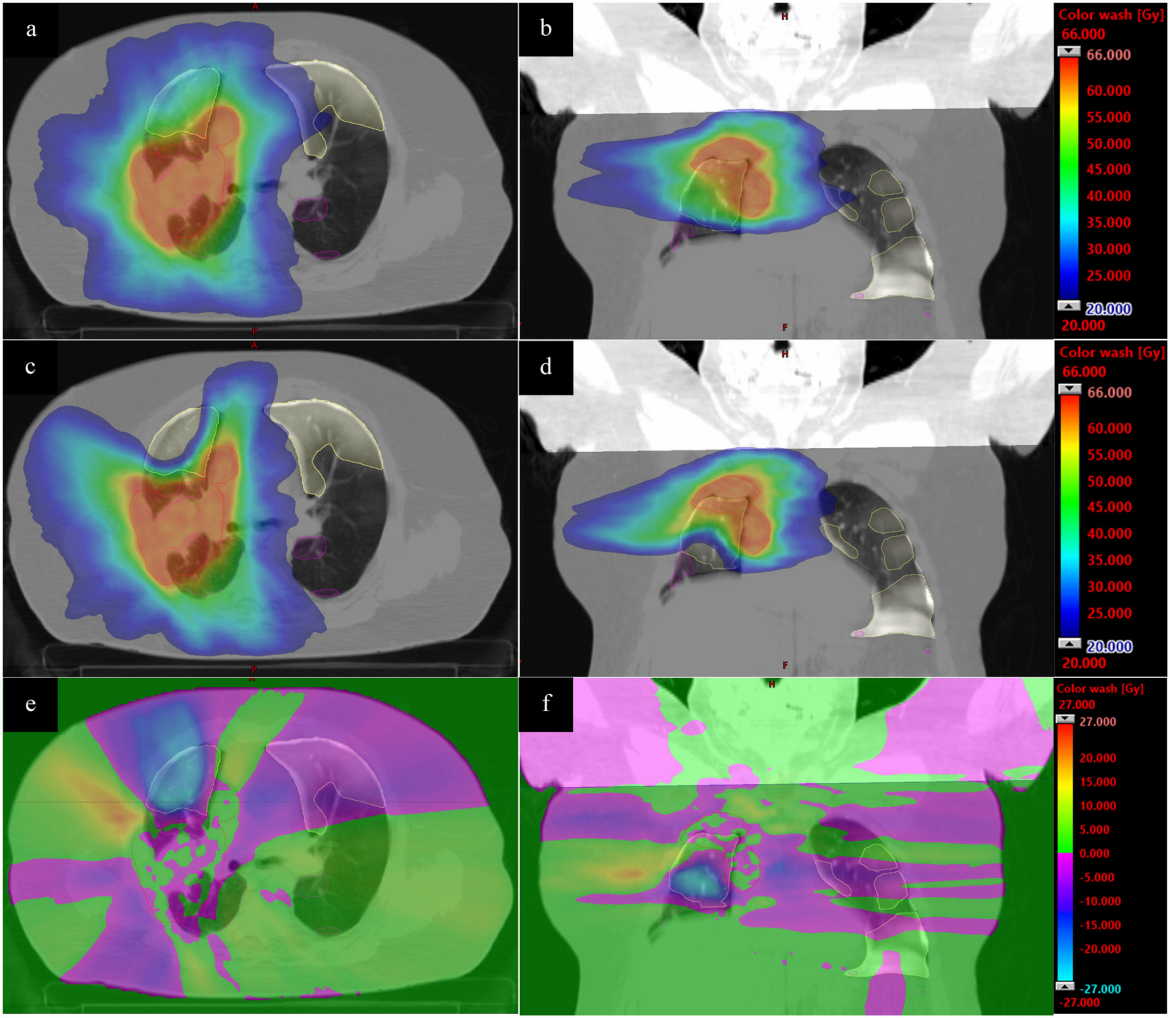
The dose distribution for the conventional plan (a, b) and parametric response mapping (PRM)–guided plan (c,d) for an example case; dose difference maps (e,f) were calculated by subtracting the conventional plan from the PRM‐guided plan, meaning a negative value signifies a lower value in the PRM‐guided plan. The PRM‐guided treatment showed a large amount of redistribution of the dose away from the small airway disease (yellow contours) regions in both lungs and the parenchymal disease (magenta contour) region in the inferior ipsilateral lung to the fat and tissue distal to the right lung and the spinal cord

**FIGURE 3:**
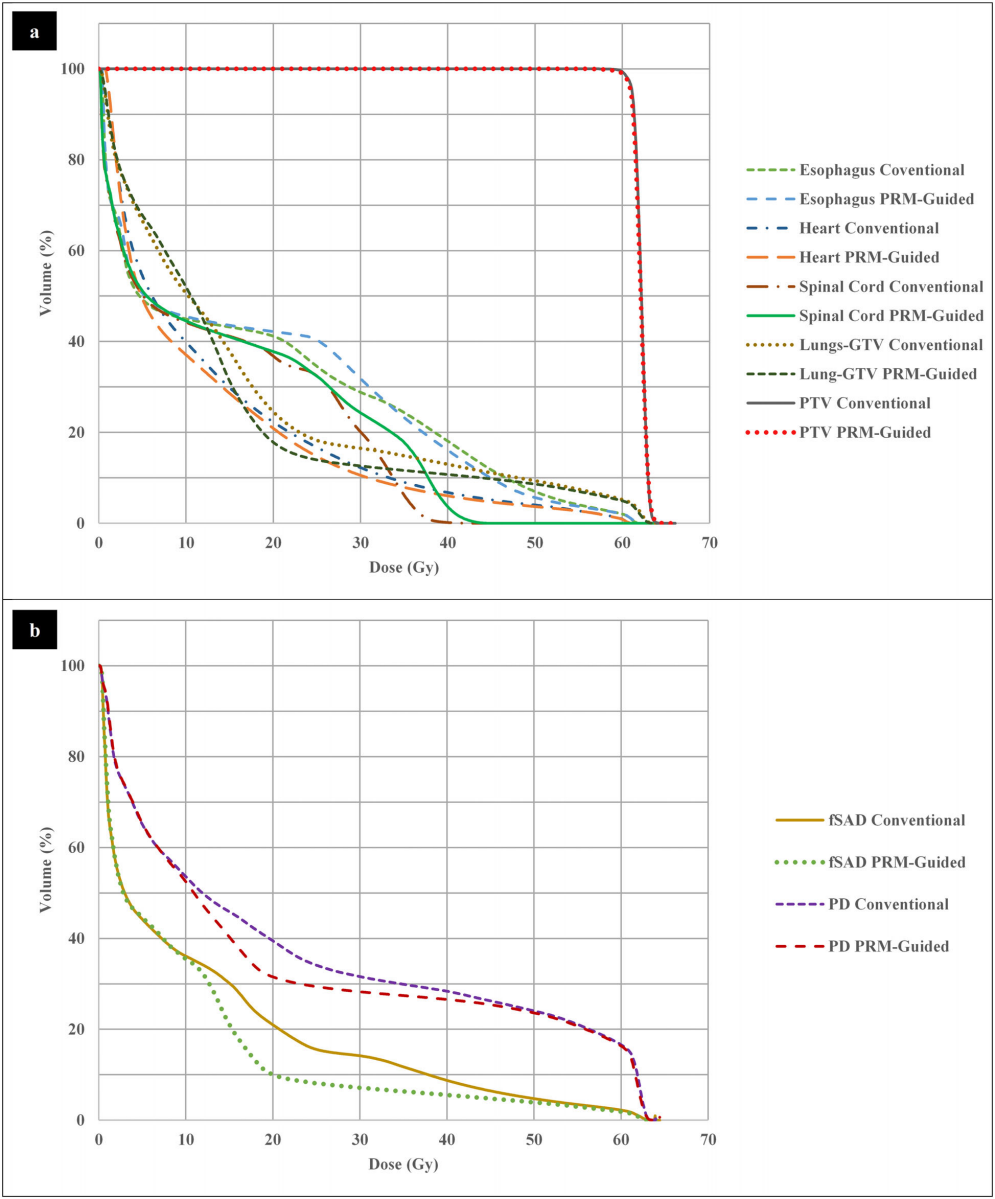
The cumulative dose volume histograms calculated for the planning target volume and organs at risk (a) and the parametric response mapping avoidance contours (b) for the example case shown in Figure 2

An example case that showed a large amount of dose redistribution is shown in Figure 2. Comparisons of the contour DVHs for this case are shown in Figure 3. In the conventional plan, the 20 Gy‐isodose regions encompassed almost the entire SAD and PD regions in the ipsilateral lung and a portion of the SAD region in the contralateral lung. Inclusion of PRM guidance caused the optimizer to redistribute dose from the anterior SAD region to the region of fat and muscle distal to the lung and to the spinal cord, resulting in a V20Gy(%) decrease of 10.95% and 7.94% for the SAD and PD contours, respectively. The lungs‐GTV V20Gy(%) decreased by 6.73% due to this redistribution, but came at the acceptable cost of a 2.77 Gy spinal cord D0.03cc increase and 2.57% decrease in PTV DC0.1cc. This also resulted in an 18.9% increase in the aperture‐based edge metric in the PRM‐guided plan.

## 4 DISCUSSION

In this study, PRM guidance was implemented into a clinical treatment planning workflow and retrospectively utilized for lung cancer patients. Overall, the optimizer was effective at implementing the PRM data into the cost function. In a metanalysis performed by Bucknell et al., it was found that the improvement of V20Gy(%) for a function‐guided treatment plan compared to a conventional plan was 4.2% (95% CI: 2.3:6.0) on average for 12 publications from literature using a variety of functional lung imaging modalities.^6^ The average V20Gy improvements of the PRM contours in this study, 5.4%, compared well to the literature. Note that the 18 patients in this work were not screened before the study based on the feasibility of effective functional‐guided treatment planning.

Another encouraging result of this study was that the inclusion of PRM guidance in treatment planning did not redistribute the dose to other sensitive organs. In the case of the lungs‐GTV, redistribution of the dose away from the PRM avoidance contours was found to decrease the V20Gy and mean dose, as seen in Table 3. This suggests that the PRM‐guidance did not redistribute the dose to normal lung regions, but instead distributed the 20 Gy dose outside of the lung entirely to regions of less sensitive fat or muscle. The increased plan complexity and MUs due to function guidance was consistent with literature.^34^, ^35^, ^36^

During this work, the reduction of the 20 Gy‐dose volume in the regions of high volume densities of PRM‐defined lung abnormalities was used for the purpose of testing the PRM‐guided treatment planning workflow and the bounds for which the dose could be redistributed within the lung. This pulmonary abnormality‐avoidance technique was based on the recent work of Owen et al. and Otsuka et al. In the work by Owen et al., a retrospective study of 88 non–small cell lung cancer radiotherapy patients with pretreatment ventilation and perfusion (VQ) SPECT imaging investigated which dose‐function metrics were most associated with a risk of RILT. This analysis found that based on receiver operating characteristic (ROC) analysis, the best predictor of RILT was the combined VQ low‐functioning lung receiving at least 20 Gy.^15^ In a similar investigation, Otsuka et al. retrospectively studied 40 thoracic cancer patients treated with radiotherapy that had pretreatment 4DCT data. They determined that the best predictors of RILT based on ROC analysis were the mean dose, V20Gy, and V5Gy within the poorly ventilated regions.^18^ It is important to reiterate that during this current work, regions of 20 Gy dose in the abnormal function PRM contours were not funneled to other regions of the lung that had more normal function, as previously described, which is shown by the significant V20Gy and mean dose decreases in Table 3. Also, one could feasibly customize the workflow from this study to avoid regions of normal lung function. This could be achieved by reducing the V20Gy to regions of high PRM‐defined normal lung classification volume density or by creating a contour that is the subtraction of the high densities of abnormal function from the lungs‐GTV contour.

An advantage of the PRM‐guided workflow presented in this work is its potential as a relatively straightforward tool for the screening of patient lung function and the guidance of treatment plans accordingly. Since the calculation of PRM classification is based on paired inspiration and expiration CT scans, this could be easily accomplished on any modern CT simulator, making this technology more readily available for smaller clinics without additional patient appointments. The creation of TPS plugins to assist in CT data registration and PRM classification could minimize the additional work required when utilizing PRM guidance and keep the analysis within the TPS, reducing data transfer errors. One potential method to reduce the workload further would be to use the mapping of PRM as a screening tool to triage which patients could benefit from PRM‐guided planning. During this study, the patients that generally showed the largest improvements in PRM contour V20Gy were the patients who had reduced overlap between targets and the avoidance contours with less portions of the avoidance contours surrounding the target. This was consistent with the work of Munawar et al.^8^ Clinicians could potentially identify patients with these traits after the registration of the PRM expiration CT and the treatment planning CT and use these data to determine whether the patient would benefit from PRM‐guided treatment optimization. If patients would likely not benefit from PRM guidance, the data could be excluded to avoid any unnecessary increases in plan complexity. This decision making process could be further assisted with feasibility analysis tools.^37^

It is important to note that although the use of PRM guidance did reduce the V20Gy of the avoidance contours, the clinical impact of PRM‐guided treatment planning is unknown. To the authors’ knowledge, this work is the first application of PRM to radiotherapy treatment planning and no clinical trials have been performed investigating its clinical impact. Other methods of CT‐based function‐guided treatment planning have begun to undergo clinical trials, with results of a prospective clinical trial showing the potential to reduce grade 2 and 3 pneumonitis incidence in patients who received function‐guided treatment.^11^

Although PRM‐guided treatment planning was shown to be effective during this work, its utilization did have some difficulties. For two of the patients who had their treatment plans created on DIBH CT scans, the lung volume in the DIBH scan was more than double the volume in the PRM expiration CT, which presented a challenge for alignment. DIR between these scans required multiple attempts with varying rigid initializations, potentially indicating that this level of motion exceeds the limitations of the Velocity algorithm. The resulting DIRs demonstrated increased error in the inferior edges of the lung but the errors in this region did not appreciably impact the propagation of the PRM contours. In future implementations of this workflow, the DIR process for cases with large breathing motions could be addressed through modification of the DIR algorithm or implementation of a biomechanical algorithm better suited to handle such deformations.^38^ Another potential challenge is the quality assurance of the PRM CT scans. It is crucial for the calculation of PRM that CT scans are acquired at total lung capacity (i.e., full inspiration) and functional residual capacity or residual volume (i.e., expiration). If this does not occur, it may cause incorrect PRM classification of regions of the lung. This issue can be relatively common in patients that are severely impacted by tumor burden or lung disease, making the full inspiration and expiration breathing maneuvers difficult. Nevertheless, large multicenter observational trials, such as COPDGene^39^ and SPIROMICS,^40^ have implemented this paired CT protocol in COPD patients. The investigators of these trials have found established guidelines for the reproducibility of quantitative CT metrics, such as PRM.^22^, ^41^, ^42^

## 5 CONCLUSIONS

This study implemented PRM guidance into a clinical workflow for the radiotherapy treatment planning of lung cancer patients. PRM guidance was found to improve the V20Gy of PRM‐defined avoidance regions of the lung effectively compared to conventional treatment plans at a scale similar to other function‐guided treatment planning methods in literature. PRM guidance did not come at the significant cost of OAR avoidance or PTV coverage, but only a slight increase in plan complexity. This work assists in providing a foundation for future clinical implementation of PRM‐guided treatment planning, which will require additional studies and clinical trials to investigate the impact of reducing the dose to regions of the different PRM classifications.

## CONFLICT OF INTEREST

Charles K. Matrosic has presented research at a Radformation trade show and received a small honorarium. Craig J. Galban has a financial interest in Imbio, LLC, which has licensed PRM from the University of Michigan. Matthew Schipper has previously consulted with Innovative Analytics outside of the submitted work. Shruti Jolly was part of the advisory boards of AstraZeneca and Varian Medical Systems and was a consultant for the Michigan Radiation Oncology Quality Consortium (MROQC, funded by Blue Cross Blue Shield of Michigan) during this work. Martha Matuszak received research funding from Varian Medical Systems and was the lead lung physicist for MROQC. All the other authors have no conflict of interest to report.

## AUTHOR CONTRIBUTION STATEMENT

D. Rocky Owen, Craig J. Galban, Martha Matuszak, and Randall K. Ten Haken conceived the presented work. Shruti Jolly and Caitlin Schonewolf led the clinical trial that acquired the imaging data and provided clinical input. Charles K. Matrosic, D. Rocky Owen, Daniel Polan, Craig Galban, and Yilun Sun performed the treatment planning study and data analysis. All authors provided feedback and interpreted the data. Martha Matuszak, Shruti Jolly, and Randall K. Ten Haken supervised the work. Charles K. Matrosic, D. Rocky Owen, and Daniel Polan wrote the manuscript with input from all authors.
